# Dr. Cassius O. Jackson

**Published:** 1902-03

**Authors:** 


Dr. Cassius O. Jackson, U. of B. 1880, died at his home in Victor, N.Y., January 27, 1902, aged 47 years. His death was due to acute pneumonia after a three days' illness. Dr. Jackson, a native of Canandaigua, was one of the best known physicians in his region, was engaged in active practice until his last illness and had been a resident of Victor since the date of his graduation.

